# 

**Published:** 1872-04

**Authors:** 


### American Medical Association.

WM B. ATKINSON, Permanent Secretary, 1400 Pine Street, Southwest corner Broad Philadelphia.

The Twenty-third Annual Session will be held in Horticultural Hall, Broad Street above Spruce, on Tuesday, May 7, 1872, at 11 A. M.

RAILROADS.—Union Pacific, return free, if first-class tickets are bought, and an acknowledgement taken from the agent. Cumberland Valley, excursion tickets. Orange, Alexandria, and Manassas, half fare for return. Pittsburg, Cincinnati, and St. Louis, excursion tickets. Pittsburg, Fort Wayne, and Chicago, excursion tickets. Cleveland and Pittsburg, excursion tickets. Central Railroad of Georgia, return free. Richmond and Petersburg, return free. Wilmington and Weldon, excursion tickets one fare. Wilmington, Columbia and Augusta, excursion tickets one fare. Kansas Pacific, one and one-fifth fare for excursion. Atlanta and New Orleans Short Line (A. and W. Pt. Western, Mobile, and M. N. O., M. and Texas Railroads), return free. Western and Atlantic, excursion tickets one fare. Western Alabama, excursion tickets one fare. Evansville and Crawfordsville, excursion tickets. Lehigh Valley, excursion tickets one fare. Louisville and Nashville, excursion tickets. Memphis and Louisville, excursion tickets. North Pennsylvania, excursion tickets two-thirds fare. Pennsylvania Central, excursion tickets. Philadelphia and Erie, excursion tickets. Philadelphia, Wilmington and Baltimore, excursion tickets. Philadelphia and Reading, excursion tickets at two-thirds. Baltimore and Ohio, excursion tickets. Lake Shore and Michigan Southern, excursion tickets if forty are taken.

☞ All who desire to avail themselves of the above rates, must send to the Secretary their full names, and the names of *all* the railroads over which they must travel in coming to the session, with stamp for postage.

WM. B. ATKINSON.

SPECIAL.—Camden and Amboy, excursion tickets at $4 from New York to Philadelphia and return, if fifty tickets are taken. For this ticket, send money to Dr. A. E. M. Purdy, 123 East Thirtieth Street, New York.

From Montgomery, Ala., to Philadelphia, and return (by Tennessee) $39 80. Apply through Dr. R. F. Michel, Montgomery, Ala.

From Washington to Philadelphia and return, $6, if fifty tickets are taken

Central Pacific, half local rates.

### Books and Pamphlets Received.

Reports of the Board of State Commissioners of Public Charities of the State of New York for 1868, 1869, 1870.

Report of the Board of State Commissioners of Public Charities of the State of New York relating to the Insane, and the capacity and cost of the several State Insane Asylums, transmitted to the Legislature March 9, 1872.

Second Inaugural Address of Hon. Alexander Brush, Mayor of Buffalo, to the Common Council, Feb. 12, 1872.

Annual Report of the Board of Health to the General Assembly of Louisiana, Dec. 31, 1871.

Transactions of the Homœpathic Medical Society of the State of New York for the year 1870, Vol. VIII.

Transactions of the Eclectic Medical Society of the State of New York, for the year 1871.

Proceedings of the Twenty-first Annual Meeting of the New York State Homœpathic Medical Society, 1872.

Annual Address, delivered before the Cleveland Homœpathic Hospital College, Feb. 14th, 1872, by J. D. Buck, M. D., Professor of Psychology.

The Lens; a Quarterly Journal of Microscopy and the allied Natural Sciences, with the transactions of the State Microscopical Society of Illinois. Edited by S. A. Briggs.

Catalogue of Second-Hand and Valuable Medical and Surgical Works, for sale by James Campbell, 18 Tremont street, Boston, Mass.

Report of John M. Woodworth, Supervising Surgeon U. S. Marine Hospital Service.

Case of Bright's Disease of the Kidneys, determined by the Ophthalmoscope. By Ruben Vance, M. D., New York.

Injuries of Nerves and their consequences. By S. Weir Mitchell, M.D., etc. Philadelphia: J. B. Lippincott & Co., 1872. Buffalo: Breed, Lent & Co.

Lectures on Aural Catarrh; or the commonest forms of deafness and their cure. By Peter Allen, M. D. New York: Wm. Wood & Co., 1872. Buffalo: Breed, Lent & Co.

The Treatment of Venereal Diseases; a monograph on the method pursued in the Vienna Hospital, under the direction of Prof. Von Sigmund. By M. H. Henry, M. D. New York: Wm. Wood & Co., 1872. Buffalo: Breed, Lent & Co.

Lithotomy and Lithotrity, illustrated, by cases in the practice of Gordon Buck, M. D., Surgeon to New York and Presbyterian Hospitals. New York: Wm. Wood & Co., 1872. Buffalo: Breed, Lent & Co.

THE BUFFALO

### Medical and Surgical Journal

PUBLISHED MONTHLY,

**Containing Original Articles, Reports of Medical Societies and Hospitals, Editorial, Reviews, Correspondence, Army News, etc., etc.; including the usual variety of Medical Periodical Publications. Specimen copies sent on application.**

Address Buffalo Medical & Surgical Journal, Buffalo, N. Y.

**TERMS OF SUBSCRIPTION, - - $3.00 PER YEAR, IN ADVANCE**

